# Prevalence and characteristics of suicidal ideation among 2199 elderly inpatients with surgical or medical conditions in Taiwan

**DOI:** 10.1186/s12888-018-1981-7

**Published:** 2018-12-22

**Authors:** Su-Jung Liao, Bo-Jian Wu, Tse-Tsung Liu, Chao-Ping Chou, Jiin-Ru Rong

**Affiliations:** 1 Department of Nursing, Ministry of Health and Welfare, Yuli Hospital, 448 Chung-Hua Road, Yuli Township, Hualien County 981 Taiwan, Republic of China; 2 Department of Nursing, National Taipei University of Nursing Health Science, No.365, Mingde Rd., Beitou Dist., Taipei City, 112 Taiwan, Republic of China; 3 Department of Psychiatry, Ministry of Health and Welfare, Yuli Hospital, 448 Chung-Hua Road, Yuli Township, Hualien County 981 Taiwan, Republic of China; 4 Department of Geriatrics, Mennonite Christian Hospital, 44, Minquan Rd., Hualien City, Hualien County 970 Taiwan, Republic of China; 5 Department of Psychiatry, Mennonite Christian Hospital, 44, Minquan Rd., Hualien City, Hualien County 970 Taiwan, Republic of China

**Keywords:** Elderly, Inpatients, Suicidal ideation, Wish to die, Quality of life, BSRS-5

## Abstract

**Background:**

Worldwide, the elderly are at a greater risk of suicide than other age groups. There is a paucity of research exploring risk factors for suicide in hospitalized elderly patients. Therefore, a study designed to explore the prevalence and characteristic of suicidal ideation (SI), such as QOL (quality of life), a wish to die (WTD), and other factors in elderly inpatients with medical or surgical conditions in Taiwan was warranted.

**Methods:**

A total of 2199 hospitalized elderly patients over age 65 were enrolled. Demographic data, 5-item Brief Symptom Rating Scale (BSRS-5), and the World Health Organization Quality of Life-BREF (WHOQOL-BREF) data were collected. Logistic regression models were used to find the SI-related factors for all participants and to investigate the covariates correlated with WTD in patients with SI. Receiver operating characteristic (ROC) curve analysis was used to find the most important items of the BSRS-5 predictive of SI in this population.

**Results:**

SI was found in 3.1% (68/2199) of the elderly. The statistically significantly factors associated with SI were: BSRS-5 item 2 (depression) (odds ratio [OR] = 2.15, 95% confidence interval [CI] = 1.56–2.98), item 4 (inferiority) (OR = 1.62, 1.23–2.13), item 5 (insomnia) (OR = 1.52, 1.13–2.05), and physical domain of WHOQOL (OR = 0.84, 0.72–0.99). QOL15 (mobility) (OR = 0.64, 0.46–0.90) and QOL 16 (satisfaction with sleep) (OR = 0.62, 0.44–0.88) were also significantly associated with SI. The status of living alone (OR = 4.44, 1.24–15.87), QOL 26 (absence of negative feeling) (OR = 0.38, 0.15–0.98), and QOL 27 (being respected/accepted) (OR = 0.43, 0.20–0.92) were significantly associated with WTD among inpatients with SI. The ROC curve analysis revealed that depression, inferiority, and insomnia were the most important items in the BSRS-5 significantly associated with SI among the elderly inpatients.

**Conclusion:**

To provide physical recovery and maintain mental health for physically ill elderly inpatients, setting up a multi-faceted approach targeting the aforementioned determinants of SI and WTD for reducing the risk of suicide attempt, and exploring other factors correlated with suicidal behaviors, are important topics and directions for clinical practice and further research.

## Background

The associated risk factors and prevalence for suicidal behaviors are diverse and are closely related to setting, measures, age groups, and different populations [1]. When compared with younger people, elderly adults are at a higher risk of suicide in most countries [2]. Concerning the risk of suicide, suicidal ideation (SI) usually plays an important role and paves the way to suicidal behaviors [3]. The prevalence of SI varies among the elderly, ranging from 0.7% in elderly primary care patients [4] to 26% in acute medically ill elderly inpatients [5].

Several studies have revealed multi-domain factors related to SI or suicidal behaviors. Quality of life (QOL) has been found to be associated with the risk of SI or suicidal behaviors in the elderly [6, 7]. In psychological autopsy, most of the cases with suicide death had a wish to die (WTD) [8], which was also found to be associated with all-cause mortality during five-year follow up in elderly primary care patients [9]. Furthermore, there are other risk factors related to SI and suicidal behaviors, which include clinical depression [5, 10–14], substance misuse [13, 15, 16], poor perception of health [11, 17], financial problems [12, 14], relationship problem [11, 14], poor social support [13, 15], living alone [18], marital status [15, 18], impaired cognition [19, 20], history of traumatic events [1], and the burden of physical illness [21].

More attention should be paid to elderly inpatients with physical illness because they are more likely to have a suicide attempt and suicide death because of old age, burden of physical diseases, and an increased concurrence of depression [5]. Thus, to single out elderly inpatients with SI at an early stage and provide timely adequate treatment in a general hospital, may decrease rates of suicidal behaviors and related mortality [5, 22]. Although this issue is important, only a small number of studies have explored the prevalence and characteristics of SI in elderly inpatients with medical or surgical conditions. One study in Iran revealed a high rate of SI in 650 hospitalized physically ill elderly patients, among whom 21.6% expressed SI, and 14.9% had a moderate to strong WTD [1]. In that study, regression models revealed that the presence of SI was significantly related to the history of traumatic events, length of hospital stay, severity of depression, and the level of social support. Another study in the UK also reported that a total of 36% had suicidal thoughts, and 22% expressed a WTD in 55 elderly patients who were admitted due to acute medical conditions [5]. Pessimism, previous deliberate self-harm, the severity of depression, and use of antidepressant were found to be significantly associated with the SI in the univariate analysis. There were some limitations in these prior studies: (1) a small sample size and use of univariate analysis to investigate SI-associated factors [5]; (2) a lack of exploration of QOL [1, 5], which seemed to play a role in SI among the elderly [6, 7]; (3) WTD and its related factors among inpatients with SI was yet to be explored; (4) there seems to be very few similar studies focusing on physically ill hospitalized elderly in the Far East, let alone in Taiwan.

Regarding the instruments for screening SI, the Department of Health in Taiwan has been using the 5-item Brief Symptom Rating Scale (BSRS-5), which has been found to present with good psychometric properties to identify psychiatric morbidity in medical settings or the community [23], for suicide prevention programs on a large scale [24, 25]. The BSRS-5, which was designed for the early detection of minor mental disorders associated with depression and anxiety, is also a very useful tool to detect the presence of SI [23, 24]. It has been used by some hospitals for routinely screening inpatients with medical illness and people receiving regular physical check-ups. The proposed cut-off score of 5/6 for a total score of 5 items of the BSRS-5, which we called “model of 5/6 for BSRS-5” hereafter, was implemented to identify people with psychiatric comorbidity [26, 27]. In a case-control study for exploring suicide attempts in the elderly, those with BSRS-5 total scores greater than 5 were 17.8 times more likely to have a suicide attempt than those with scores less than or equal to 5 [10]. A study recruiting hospitalized medical inpatients in Taiwan found that depression, inferiority, insomnia, and hostility in BSRS-5 were significantly related to the presence of SI [24]. However, in that study, age distribution, such as mean, standard deviation or percentage across age groups was not specified, and subgroup analyses focusing on the elderly or comparisons between different age groups were not done. Hence, certain items of BSRS-5 in hospitalized elderly with medical or surgical conditions, remains unknown. Consequently, two important questions related to the BSRS-5 studies are worth exploring, i.e., (1) which items of BSRS-5 are predictive of SI in the physically ill elderly inpatients, and (2) is the predictive ability of a model made up of certain items of BSRS-5 in question 1 better than that of a model of 5/6 for BSRS-5? These answers may elucidate the most important factors related to SI for physically ill elderly inpatients and set up a parsimonious model to predict SI and build up more effective strategies mainly focusing on these characteristics to diminish rates of subsequent suicidal behaviors.

Thus, the aim of this study were: (1) to explore the prevalence of SI for physically ill elderly inpatients; (2) to find the association between QOL and SI; (3) to investigate the prevalence and the factors related to WTD; (4) to explore the association between the items of BSRS-5 and SI; (5) to find a parsimonious model predictive of SI from three models using items of the BSRS-5 as predictor variables, i.e., certain items of BSRS-5 significantly associated with SI obtained from multivariate regression models, a single variable of BSRS-5 total scores greater than 5 (model of 5/6 for BSRS-5), and all five items of the BSRS-5.

## Method

### Study design and subjects

Since 2007, for maximizing the nationwide use of BSRS-5, which was called “Mood Thermometer” as a tool for mental health and suicide screening in Taiwan, the Taiwan Department of Health has been proposing a multi-site suicide prevention program with sufficient grants to hospitals. The suicide prevention program required that each grant-aided hospital should submit a research project using BSRS-5 alone rather than other SI screening instruments such as Columbia Suicide Screening [28] or Beck Scale for suicidal ideation (BSSI) [29] to single out community residents or patients with SI; subsequently, the program demanded that each subsidized hospital should refer the interviewees whose BSRS total scores were greater than 5 or those with the presence of SI to psychiatrists for further treatment. This study was a cross-sectional design. It was aided by grants and was conducted between February and December 2012 in Mennonite Christian Hospital in Hualien County, which is a referral hospital for a large area in the east of Taiwan. Subjects were selected by a convenience sampling from various medical services. A total of 2199 participants who were screened from a large pool of 2300 hospitalized elderly patients participated in this study. All of 101 patients were excluded because of reasons as follows: using antidepressants (*n* = 54, 53.4%), refusing to participate (*n* = 19, 18.8%), being admitted to wards which did not belong to the units defined in the inclusion criteria (*n* = 11, 10.9%), patients with acute critical conditions unfitted for participation (*n* = 9, 8.9%), schizophrenia patients admitted to the psychiatric ward (*n* = 8, 8%). Information was obtained from both the patients and families on the third day after admission by well-trained interviewers. Inclusion criteria included: (1) patients who were admitted to medical or surgical wards other than oncology, hospice, psychiatric wards or intensive care units; (2) patients over age 65; (3) patients who were able to communicate with interviewers. Exclusion criteria included: (1) patients who used antidepressants or mood stabilizers before admission; (2) patients had difficulty in communication due to sensory problems, or had significant cognitive impairment such as delirium or dementia. Sociodemographic information was obtained regarding sex, age, educational level (uneducated, primary school, junior high school, senior high school, or above), marital status (married, unmarried, which included single, divorced, or widowed), living situation (living alone, living with friends, or living with relatives), economic status (good, fair, or poor), and perceived health status (good or poor). The study design was reviewed by the Institutional Review Board (IRB) of Mennonite Christian Hospital. All the subjects provided informed consent before this study began.

### Measures

#### Definition of suicidal ideation

A question in BSRS-5, “Do you have any suicide ideation?” was supplemented at the end of the questionnaire. If the subject answered “Yes”, then the presence of SI was confirmed.

#### Quality of life

To measure the QOL, the Taiwanese version of the World Health Organization Quality of Life-BREF (WHOQOL-BREF TW) [30] was used to assess the global QOL of patients in medical settings. The WHOQOL-BREF TW includes 26 items (24 items that represent each of the 24 specific facets of the WHOQOL-100 and 2 global/general items). In addition, in the WHOQOL-BREF TW, two additional national items were generated and validated from the Taiwan version of the WHOQOL-100 [31]. The factor structure of the WHOQOL-BREF TW includes 4 domains, i.e., physical (QOL-PHY), psychological (QOL-PSY), social relationships (QOL-SR), and environmental (QOL-ENV). For a given item or domain of the WHOQOL-BREF, a higher score indicates a greater level of quality of life.

#### Wish to die

For those who had SI, each one should be assessed for the presence of a WTD, which was obtained using item 2 of the Beck Scale for SI (BSSI) that is a 19-item rating scale designed to evaluate the risk of suicidal intention [29]. We redefined item 2 in the BSSI, in which no wish or a weak WTD were categorized into a faint WTD (coded as 0), and a moderate or a strong WTD (MTS-WTD, coded as 1).

#### BSRS-5

The severity of distress and psychopathology were measured using the BSRS-5 [23] derived from the SCL-90-R [32]. The participants were asked to rate symptoms on a 5-point scale: 0, not at all; 1, a little bit; 2, moderately; 3, quite a bit; and 4, extremely, and a total score was calculated for each participant. A higher BSRS-5 total score indicates poorer mental health. The full scale included the following five items of psychopathology: (1) BSRS-5 item 1(anxiety): feeling tense or keyed up; (2) item 2 (depression): feeling low in mood; (3) item 3 (hostility): feeling easily annoyed or irritated; (4) BSRS-5 item 4 (inferiority): feeling inferior to others; and (5) item 5 (insomnia): having trouble falling asleep. A variable of a total score of BSRS-5 greater than 5 was formed because it represented significant psychiatric morbidity for an individual [26, 27], and it was highly associated with suicidal behavior in the elderly [10].

Cognitive function was assessed based on a Chinese version [33] of the Mini-Mental State Examination (MMSE) [34], in which the maximum score is 30, and a higher score indicates better cognition. Impaired cognition was defined for those who were uneducated and had MMSE scores less than 14, and the educated with the scores less than 24 [33].

### Statistical analyses

SPSS version 19 (IBM company) was used to conduct statistical analyses. The significance level was set at a value of 0.05 (two-tailed). The rule for the selection of covariates in multivariate logistic regression models was as follows: for avoiding multi-collinearity, the covariates selected to be placed in the regression models in this study should be tested with the analysis of correlation in advance; Spearman rho for either or both of the covariates which were categorical variables or Pearson correlation coefficient for both which were continuous variables, were obtained. If the value of coefficients between two certain explanatory covariates was greater than 0.7, then the correlation coefficient between the dependent variable with these two explanatory covariates was examined. The covariate with greater absolute value of correlation coefficient between it and the dependent variable was put into the regression model.

#### Model 1: Multivariate logistic regression models to explore factors related to SI

For the reduction in the number of variables in the regression models, the univariate analyses were firstly used to explore the association between variables of interest and SI. Subsequently, the variables with statistically significant differences between groups were put into two multivariate logistic models. In the following description of different models, “A” denoted that “some or all items of BSRS-5” were included in the model, and “B” indicated that the model included the variable of “BSRS-5 total score greater than 5”.

##### Model 1A

Model 1A included independent variables, such as five items of BSRS-5, age, gender, and other covariates with statistically significant differences between groups.

##### Model 1B

In Model 1B, except for the variable of BSRS-5 total scores greater than 5, which replaced the BSRS-5 item 1 through 5, other variables were the same as those in the Model 1A.

#### Model 2: Multivariate logistic models to explore the items of WHOQOL-BREF related to SI

For the reduction in the number of variables in the multivariate regression analysis, if some domains of WHOQOL-BREF were found to be statistically significantly associated with SI in Model 1A or Model 1B, then all items of these domains would be compared using a univariate analysis. The WHOQOL-BREF items with significant differences between groups were put into Model 2A and Model 2B.

##### Model 2A

The independent variable of Model 2A included the WHOQOL-BREF items with significant differences after the univariate analysis, the same demographic factors included in Model 1A, and the items of BSRS-5 significantly related to SI in Model 1A.

##### Model 2B

In Model 2B, except for the variable of BSRS-5 total scores greater than 5, which replaced the items of BSRS-5 in Model 2A, all independent variables were the same as those in Model 2A.

#### Model 3: Univariate logistic regression models exploring factors related to MTS-WTD in those who had SI

Univariate logistic regression models were used to explore the association between covariates and MTS-WTD because we expected the number of patients with MTS-WTD would be small in those who had SI. Except for the individual sum of four domains of WHOOQOL-BREF, all variables in Model 1A and 28 items of the WHOOQOL-BREF were put into univariate models as covariates.

#### Model 4: Comparison between different propensity score models of BSRS-5 predicting SI using Receiver Operating Characteristic (ROC) curve analysis

To get a parsimonious model predicting SI, we used a propensity score model (PSM), in which multiple variables were included and formed a propensity score (PS) (the value was between 0 and 1); the coefficients of covariates of the PSM were obtained from a multivariate logistic model [35]. The PS generated from different models was used as a state variable in the ROC curve analysis.

##### Model 4A

The state variable in Model 4A was the PS generated from the PSM, which included certain BSRS-5 items found to be significantly associated with SI in Model 1A as predictor variables.

##### Model 4B

Model 4B, i.e., the “model of 5/6 of BSRS-5”, included a state variable, which was the PS generated from the PSM, and the covariate of BSRS-5 total scores greater than 5 as a predictor variable.

##### Model 4C

In Model 4C, the state variable was the PS generated from the PSM, which included all items of BSRS-5 as predictor variables.

The difference in areas under the curve (AUCs) between each two models was compared [36]. We think a good model predicting SI should meet the following conditions: (1) the sensitivity, specificity, positive predictive value (PPV), negative predictive value (NPV), and accuracy, i.e., five indexes of ROC should be all greater than or equal to 0.8; (2) If two models met criteria 1, and the difference in AUCs between them did not reach a statistically significant level, then a parsimonious model was selected.

## Results

### Demographic data

The average age for all participants (*n* = 2199) was 76.4 ± 7.4. Male patients accounted for 55.4% (*n* = 1218). A total of 1195 (54.5%) were admitted to medical wards, in which most received the treatment from the team of pulmonary medicine (*n* = 276, 12.5%). For patients admitted to surgical wards (*n* = 908, 41.2%), most were under the care of general surgery (*n* = 249, 11.32%). Missed classification of ward accounted for 4.36% (*n* = 96). Table 1 presents the classification of subspecialty teams which took care of patients admitted to the general hospital. SI was found in 3.1% (68/2199) of the elderly, and 25.3% (557/2199) had BSRS-5 total scores greater than 5. Approximately one-fourth of participants with SI had a MTS-WTD (25.5%, 14/68). Sociodemographic variables are presented in Table 2. Table 3 shows the results of the comparison of characteristics between those with SI and those without. There were statistically significant differences between the two groups in variables as follows: being unmarried, having poor economic status, perception of poor health, BSRS-5 item 1–5, BSRS-5 total scores greater than 5, physical domain of WHOOQOL-BRIEF(QOL-PHY), psychological domain of WHOOQOL-BRIEF(QOL-PSY), and environmental domain of WHOOQOL-BRIEF(QOL-ENV). Table 4 shows the illustration of different models in this current study.

**Table 1:** Classification based on medical or surgical wards for participants admitted to the general hospital (*n* = 2199)

Classification of ward	Team	n	Percentage
Medical ward	–	1195	54.3%
	Cardiology	167	7.59%
	Nephrology	233	10.59%
	Neurology	206	9.36%
	Gastroenterology	247	11.23%
	Pulmonary medicine	276	12.55%
	Infectious disease	66	3.0%
Surgical ward	–	908	41.29%
	Neurosurgery	100	4.54%
	Urology	245	11.14%
	Orthopedics	235	10.68%
	Plastic surgery	79	3.59%
	General surgery	249	11.32%
Missed classification		96	4.36%

**Table 2:** Participant characteristics and demographic data

Variables		Minimum	Maximum	Mean	S.D.
Age (years)	–	65	101	76.4	7.4
Sex (n, %)	Male	–	–	1218	55.4
Marital status (n, %)	unmarried	–	–	1038	47.2
Education (n, %)	Uneducated	–	–	849	38.6
	Primary school	–	–	374	17.0
	Junior high school	–	–	688	31.3
	Senior high school or above	–	–	288	13.1
Living alone (n, %)	Alone	–	–	425	19.3
Economic status	Poor	–	–	341	15.5
(n, %)	Fair	–	–	1671	76.0
	Good	–	–	187	8.5
Perception of health status (n, %)	Poor	–	–	1281	58.3
Suicide ideation (n, %)		–	–	68	3.1
BSRS	Total scores	0	17	3.7	3.0
BSRS total score > 5 (n, %)		–	–	557	25.3
Quality of life subdomains	Total scores	26.4	77.4	52.4	7.3
	Physical health	5.1	19.4	12.5	2.5
	Social relationship	6.0	20.0	13.5	2.2
	Environmental	5.8	19.1	13.7	1.9
	Psychological	6.0	19.3	12.9	2.3
MMSE	Total scores	0	30	21.1	6.2
Impaired cognition (n, %)		–	–	850	38.7

Non-living alone: living with people including spouse, children, relatives, friends

Unmarried: current marital status including single, divorced, windowed

*BSRS* Brief Symptom Rating Scale

*MMSE* Mini-Mental State Examination

**Table 3:** A comparison of characteristics between those with suicidal ideation and those who were without

Variables	Suicidal ideation	Non-suicidal ideation	*p*-value
Age (years)	76.0 (7.9)	76.4 (7.3)	0.68
Sex, male (n, %)	32 (47.1)	1186 (55.7)	0.16
Education (n, %)			0.23
Non-educated	30 (44.1)	819 (38.4)	
Primary school	12 (17.6)	362 (17.0)	
Junior high school	14 (20.6)	674 (31.6)	
More than senior high school	12 (17.6)	276 (13)	
Unmarried (n, %)	41(60.3)	997 (46.8)	0.03
Living alone (n, %)	17 (25.0)	408 (19.1)	0.23
Good economic support (n, %)	40 (58.8)	1395 (65.5)	0.26
Economic status (n, %)			0.001
Poor	25 (36.8)	316 (14.8)	
Fair	40 (58.8)	1631 (76.5)	
Good	3 (4.4)	184 (8.6)	
Perception of poor health (n, %)	56 (82.4%)	501 (23.5)	0.001
BSRS total score greater than 5 (n, %)	12 (17.6%)	1630 (76.5%)	0.001
BSRS-5 item 1 (anxiety)	1.54 (1.11)	0.67 (0.89)	0.001
BSRS-5 item 2 (depression)	2.19 (0.93)	0.87 (0.95)	0.001
BSRS-5 item 3 (hostility)	1.66 (1.16)	0.62 (0.85)	0.001
BSRS-5 item 4 (inferiority)	1.04 (1.2)	0.20 (0.56)	0.001
BSRS-5 item 5 (insomnia)	2.09 (1.04)	1.14 (1.01)	0.001
QOL-physical health	10.35 (1.19)	12.53 (2.46)	0.001
QOL-psychological	11.06 (1.83)	12.85 (2.2)	0.001
QOL-social relationship	13.07 (2.04)	13.54 (2.15)	0.07
QOL-environmental	12.51 (1.88)	13.63 (1.87)	0.001
Impaired cognition	21 (30.9%)	38.9 (38.9%)	0.18

Comparison analyses were used to compare the difference in variables between those with suicidal ideation and those without using independent t-test or chi-square test

Unmarried: current marital status including single, divorced, widowed

*BSRS* Brief Symptom Rating Scale, *QOL* quality of life rated with the WHOQOL-BREF

**Table 4:** Illustration of different models in this study

Model 1: Multivariate logistic regression models exploring factors related to SI	1A: Independent variables included age, gender, economic status, perception of poor health, unmarried, QOL-PHY, QOL-PSY, and five items of BSRS-5 item.
	1B: Independent variables included: variables were the same as those in the Model 1A except for the variable of BSRS-5 total scores greater than 5, which replaced the BSRS-5 item 1 through 5.
Model 2: Multivariate logistic models exploring the items of WHOQOL-BREF related to SI	2A: Independent variables included BSRS-5 item 2 (depression), item 4 (inferiority), item 5 (insomnia), and all items of QOL-PHY except QOL11 (feeling of bodily appearance) and QOL17 (level of daily activities).
	2B: Independent variables: all were the same as those in Model 2A except for the variable of BSRS-5 total scores greater than 5 replacing BSRS-5 item 2 (depression), item 4 (inferiority), item 5 (insomnia).
Model 3: Univariate logistic regression models exploring factors related to MTS-WTD in those who had SI	Independent variables: variables in Model 1A and 28 items of the WHOOQOL-BREF except for the individual sum of four domains of WHOOQOL-BREF, i.e., QOL-PHY, QOL-PSY, QOL-ENV, QOL-SR
Model 4: Comparison between different propensity score models of BSRS-5 predicting SI using ROC curve analysis.	State variable in 4A-4C: propensity score from individual propensity score model
	4A: predictor variables included BSRS-5 item 2 (depression), item 4 (inferiority), and item 5 (insomnia)
	4B: predictor variable: the variable of BSRS-5 total scores greater than 5
	4C: predictor variables included all five items of BSRS-5

*SI* suicidal ideation, *BSRS* Brief Symptom Rating Scale, *WHOQOL-BREF* the World Health Organization Quality of Life-BREF, *QOL-PHY* physical domain of WHOQOL-BREF, *QOL-PSY* psychological domain of WHOQOL-BREF, *QOL-ENV* environmental domain of WHOQOL-BREF, *QOL-SR* the domain of social relationships of WHOQOL-BREF, *QOL* quality of life rated with the WHOQOL-BREF, *MTS-WTD* moderate to severe wish to die, *ROC* Receiver Operating Characteristic

### Model 1: Factors related to SI in the multivariate analysis

The correlation analysis found that QOL-PSY and QOL-ENV was highly correlated (Pearson correlation coefficient = 0.74). Finally, QOL-ENV was deleted because the Spearman rho coefficient between QOL-PSY and SI was − 0.139, the absolute value of which was greater than that between QOL-ENV and SI (Spearman rho coefficient = − 0.09). In addition to age and gender, all those variables with statistically significant differences between those with SI and those without in Table 3 were put into regression models. Table 5 shows the factors statistically significantly related to SI in different models.

**Table 5:** Multivariate logistic regression models exploring factors related to suicidal ideation

Independent variables	Model 1A^a^		Model 1B ^b^	
	AOR	95% CI	AOR	95% CI
Age	1.002	0.96–1.04	0.98	0.94–1.02
Gender	1.11	0.63–1.93	0.99	0.58–1.70
Economic status (reference level = poor)				
Fair	0.74	0.41–1.35	0.61	0.35–1.08
Good	0.6	0.15–2.29	0.57	0.16–2.04
Single	1.44	0.80–2.58	1.42	0.82–2.48
Perception of poor health	1.14	0.55–2.36	1.08	0.54–2.15
BSRS-5 total scores > 5 (ref. level: ≤ 5)	–	–	9.36^**^	4.86–18.0
BSRS-5 item 1(anxiety)	1.25	0.93–1.68	–	–
BSRS-5 item 2(depression)	2.15^**^	1.56–2.98	–	–
BSRS-5 item 3(hostility)	1.22	0.91–1.64	–	–
BSRS-5 item 4(inferiority)	1.62^**^	1.23–2.13	–	–
BSRS-5 item 5(insomnia)	1.52^**^	1.13–2.05	–	–
QOL (physical health)	0.84^*^	0.72–0.99	0.81^**^	0.69–0.94
QOL (psychological)	0.93	0.79–1.09	0.89	0.77–1.04

^*^p < 0.05; ^**^ *p <* 0.01; *AOR* adjusted odds ratio, *BSRS* Brief Symptom Rating Scale, *QOL* quality of life rated with WHOQOL-BREF

^a^Model 1A: independent variables included age, gender, economic status, perception of poor health, non-married, QOL-physical health, QOL-psychological, QOL-environment and five items of BSRS-5

^b^Model 1B: except for BSRS total score greater than 5 which replaced five items of BSRS-5, other variables were the same as those in the Model 1A

#### Model 1A

Model 1A included independent variables as follows: age, gender, economic status, perception of poor health, non-married, QOL-PHY, QOL-PSY, and five items of BSRS-5 item. Model 1A revealed factors statistically significantly associated with SI as follows: BSRS-5 item 2 (depression) (OR = 2.15, 95% CI = 1.56–2.98), item 4 (inferiority) (OR = 1.62, 95% CI = 1.23–2.13), item 5 (insomnia) (OR = 1.52, 95% CI = 1.13–2.05), and QOL-PHY (OR = 0.84, 95% CI = 0.72–0.99).

#### Model 1B

In Model 1B, BSRS-5 total scores greater than 5 (OR = 9.36, 95% CI = 4.86–18.0) and QOL-PHY (OR = 0.81, 95% CI = 0.69–0.94) were statistically significantly associated with the presence of SI.

### Model 2: Items of QOL related to SI

Table 6 shows the items of WHOQOL-BREF that were related to SI. We put items of QOL-PHY into Model 2A and Model 2B because the scores of QOL-PHY were found to be related to SI in Model 1A and Model 1B.

**Table 6:** Multivariate logistic regression models exploring items of quality of life related to suicidal ideation

Independent variables	Suicidal ideation (*n* = 68)	
Model 2A for QOL related to SI ^a^	AOR	95% CI
QOL Item 15 Mobility	0.60^**^	0.42–0.85
Model 2B for QOL related to SI ^b^		
QOL Item 15 Mobility	0.64^*^	0.46–0.90
QOL Item 16 Satisfaction with sleep	0.62^**^	0.44–0.88

^*^p < 0.05; ^**^ *p <* 0.01; *AOR* adjusted odds ratio

*SI* suicidal ideation, *QOL* quality of life rated with WHOQOL-BREF, *BSRS* Brief Symptom Rating Scale

^a^Multivariate analysis: dependent variable: suicidal ideation; independent variables included demographic factors as those in Model 1, BSRS-5 item 2 (depression), BSRS-5 item4 (inferiority), BSRS-5 item5 (insomnia) and all items of QOL-physical health except item 11 and item 17-daily activities which was highly correlated with item 15-mobility (pearson correlation coefficient = 0.73) causing multi-collinearity. Only significant QOL variables were presented in this table

^b^Multivariate analysis: except BSRS total score greater than 5 which replaced BSRS-5 item 2, BSRS-5 item 4 and BSRS-5 item 5, all independent variables were the same as those in the Model 1A. Only significant QOL variables were presented in this table

#### Model 2A

Model 2A included the same demographic factors as those in Model 1A, BSRS-5 item 2 (depression), item 4 (inferiority), item 5 (insomnia), and all items of QOL-PHY except QOL11 (feeling of bodily appearance), which did not have statistically significant associations with SI in the univariate analysis, and QOL17 (level of daily activities), which was highly correlated with QOL15 (mobility) (Pearson correlation coefficient = 0.73). The QOL17 was deleted in Model 2 because the Spearman rho coefficient between the QO15 and SI was − 0.11, the absolute value of which was greater than that between QOL 17 and SI (spearman rho coefficient = − 0.09). Model 2A revealed that QOL15 (mobility) was statistically significantly associated with SI (OR = 0.60, 95% CI = 0.42–0.85).

#### Model 2B

In Model 2B, except for the variable of BSRS-5 total scores greater than 5, which replaced BSRS-5 items 2, 4, and 5, all independent variables were the same as those in Model 2A. The Model 2B revealed that QOL15 (mobility) (OR = 0.64, 95% CI = 0.46–0.90) and QOL16 (satisfaction with sleep) (OR = 0.62 95% CI = 0.44–0.88) were statistically significantly associated with SI.

### Model 3: Factors related to MTS-WTD

Table 7 presents the factors significantly related to MTS-WTD among participants with SI. A univariate model was used rather than a multivariate model due to a small number of participants with MTS-WTD (*n* = 14), which might result in a low statistical power if a multivariate model were used. The results of Model 3 showed that living alone (OR = 4.44, 95% CI = 1.24–15.87), QOL 26 (absence of negative feeling) (OR = 0.38, 95% CI = 0.15–0.98) and QOL 27(being respected/accepted) (OR = 0.43, 95% CI = 0.20–0.92) were statistically significantly associated with MTS-WTD among inpatients with SI.

**Table 7:** Univariate logistic regression models exploring factors associated with a moderate to strong wish to die

Independent variables	Moderate to strong wish to die ^a^ (*n* = 14)	
Living alone	4.44^**^	1.24–15.87
QOL Item 26 Absence of negative feeling	0.38^*^	0.15–0.98
QOL Item 27 Being respected/accepted	0.43^*^	0.20–0.92

^*^p < 0.05; ^**^ *p <* 0.01

*QOL* quality of life rated with WHOQOL-BREF

^a^Univariate analysis: dependent variable: a moderate to strong wish to die; independent variables included all demographic factors, i.e., age, gender, economic status, economic resources, perception of poor health, single, living alone, impaired cognition, five items of the BSRS-5 and the variable of BSRS-5 total scores greater than 5 as well as QOL item 1–28

### Model 4: Comparison between different models of BSRS-5 predicting SI

Table 8 shows the results of comparison between different models predicting SI.

**Table 8:** Comparison of indexes of receiver operation characteristic curve between different models

Model	△AUC	AUC	SEN	SPC	PPV	NPV	Accuracy
Model 4A^a^	△A-C	0.88	0.83	0.80	0.81	0.83	0.82
Model 4B^b^	△A-B^*^	0.79	0.82	0.76	0.77	0.81	0.79
Model 4C^c^	△B-C^*^	0.88	0.82	0.82	0.82	0.82	0.82

*AUC* area under curve, *SEN* sensitivity, *SPC* specificity, *PPV* positive predictive value, *NPV* negative predictive value

△AUC = difference in AUC between various models

△A-B^*^: significant difference in AUC between Model 4A and Model 4B

(*p* < 0.00001); △B-C^*^: the same meaning as that of △A-B^*^ (p < 0.00001); △A-C: no significant difference in AUC between Model 4A and Model 4C (*p* = 0.785)

^a^Model 4A

Propensity model:

Y = 0.963*BSRS-5 item 2 + 0.564*BSRS-5 item 4–0.655*BSRS-5 item 5 + 6.285

*propensity score* = expY/(1 + expY);

For a given individual, if the *propensity score* > 0.0355, then the model was predictive of suicidal ideation

^b^Model 4B

BSRS-5 total score greater than 5 coded as 1, otherwise as 0

Propensity model:

Y = 2.72*(BSRS-5 total score greater than 5)-4.911

*propensity score* = expY/(1 + expY);

For a given individual, if the *propensity score* > 0.0539, then the model was predictive of suicidal ideation

^c^Model 4C

Propensity model:

Y = 1.226*BSRS-5 item 1 + 2.337*BSRS-5 item 2 + 1.297*BSRS-5 item 3

+ 1.646*BSRS-5 item 4–1.694*BSRS-5 item 5 + 6.374

*propensity score* = expY/(1 + expY);

For a given individual, if the *propensity score* > 0.0386, then the model was predictive of suicidal ideation

#### Model 4A

Model 4A used a PSM to get a PS, in which predictor variables included BSRS-5 item 2 (depression), item 4 (inferiority), and item 5 (insomnia); these three items were obtained from Model 1A. In this model, for a given subject, if the PS > 0.0355, then it was predictive of SI.

#### Model 4B

In Model 4B, i.e., the model of 5/6 for BSRS-5, for a given subject, if the PS > 0.0539, then it was predictive of SI.

#### Model 4C

Model 4C, in which predictor variables included all five items of BSRS-5, for a given subject, if the PS > 0.0386, then it was predictive of SI.

**Fig. 1:**
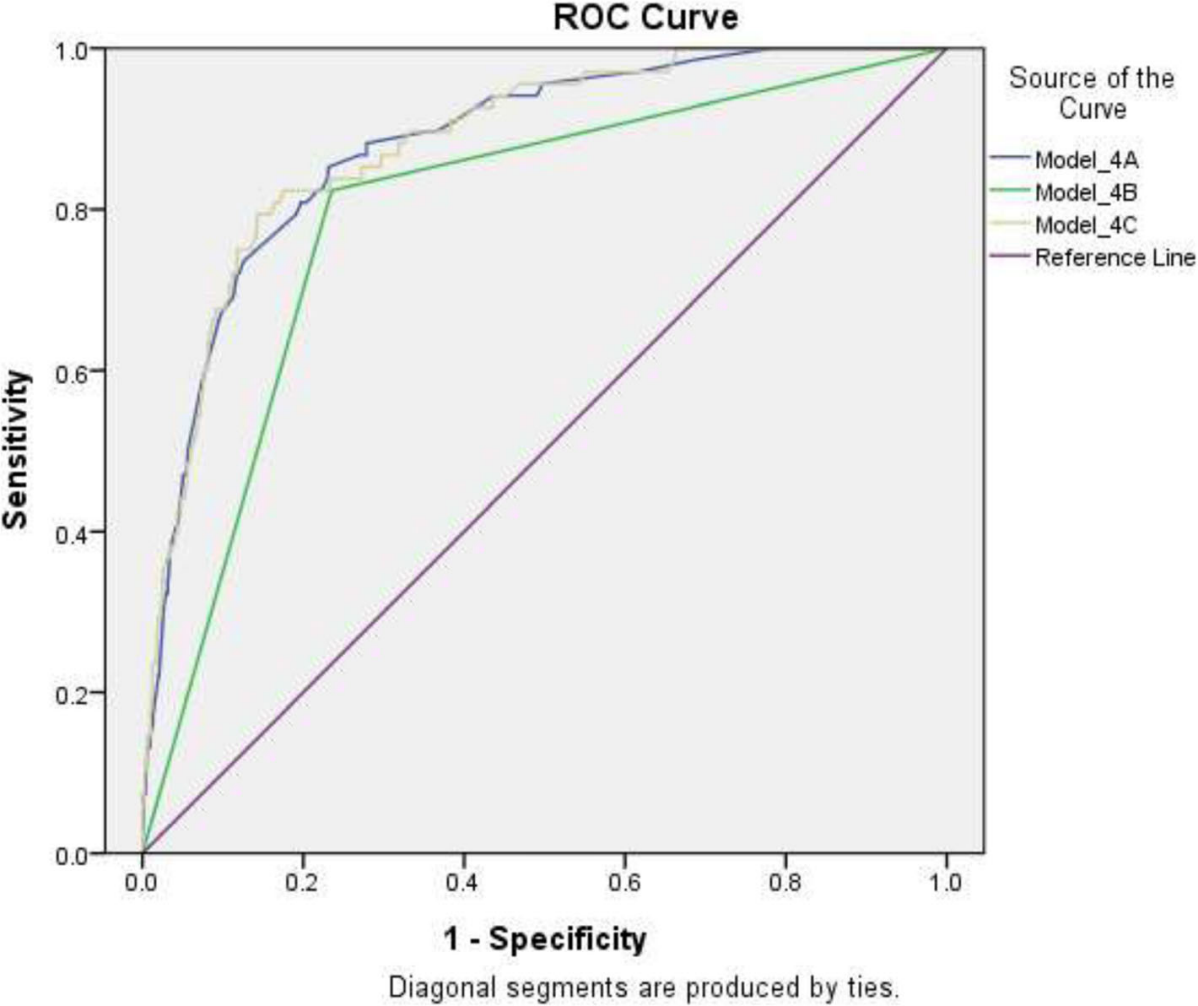
Comparison of receiver operation characteristic curve between different models. Note. State variable in 4A-4C: propensity score from individual propensity score model. Model_4A: predictor variables included BSRS-5 item 2 (depression), item 4 (inferiority), and item 5 (insomnia). Model_4B: predictor variable: the variable of BSRS-5 total scores greater than 5. Model_4C: predictor variables included all five items of BSRS-5

Figure 1 shows that the AUC of Model 4B was the least among three models. There were no statistically significant differences between the AUC of Model 4A and that of Model 4C (*p* = 0.785). However, the AUCs of both Model 4A and Model 4C were statistically significantly greater than that of Model 4B (*p* < 0.00001). Regarding the indexes of ROC, the specificity, PPV, and accuracy in the Model 4B were all below 0.8. Conversely, five indexes of ROC of Model 4A and Model 4C were all greater than or equal to 0.8. In summary, Model 4A was the parsimonious model predictive of SI. In the BSRS-5, item 2 (depression), item 4 (inferiority), and item 5 (insomnia) were the most important items significantly associated with SI among the elderly inpatients in this study.

## Discussion

### The prevalence of SI

Our study found that the 3.1% of the elderly hospitalized patients with medical or surgical conditions had SI. Notably, the prevalence of SI in the current study was much lower than those of prior studies, i.e., 36% in the UK [5] and 21.6% in Iran [1]. The inconsistency might be explained by the following reasons. First, there were differences in the instruments assessing SI, i.e., the BSSI in both studies [1, 5] vs. the item 6 of BSRS-5 in this current study. One of the former studies used the total scores of 19 items of the BSSI; SI was coded if the total score for an individual was greater than 4 [1]. However, in this study, every interviewee was asked a present-tense-question: “Do you have any suicide ideation?” The definition of SI out of yes/no is different from those of the former studies. In addition, patients in the former studies had acute physical conditions, yet in our study, participants included acute or subacute patients who might be admitted for elective procedures, such as elective gastroscopy or elective operation. Finally, the discrepancy in the prevalence might be partly accounted for by the various sample sizes among three studies, i.e., 55 [5], 650 [1], and 2199 in our study.

### Primary analysis of factors including demographic variables, BSRS-5, and four domains of QOL related to SI

Our study also found depression, inferiority, insomnia in BSRS-5, and the sum of physical domain of QOL were significantly associated with SI. As our result indicated, depression is a major determinant of SI or suicidal behavior among elderly people in the community [6, 10, 13] or inpatients [1, 5]. Although insomnia is highly associated with depression, after adjustment in the regression model, it was still significantly associated with SI. This finding is compatible with that of a study which found that poor sleep increases the risk of SI, but only among people with no or one mental health condition [37]. Total sleep time is an important predictor of suicidal behavior that requires particular attention [38], and changes in insomnia drive subsequent changes in suicidal ideation [39]. Therefore, it is important for physicians to detect insomnia as a sign of suicidal behavior, regardless of which illness a patient may suffer from [40]. Of interest, our study found that inferiority, which is seen as a lack of covert self-esteem [41], was correlated with SI in elderly inpatients. This finding is compatible with that of a study which found that low self-esteem was associated with suicidal intent, independently of the severity of depression [42]. A study recruiting 2964 community members also found that low self-esteem is one of the major risk factors related to SI [43], and another study revealed that low self-esteem and low sense of self-efficacy may lead to a suicide attempt [44]. Two other studies in elderly inpatients [1, 5] revealed that factors related to SI were use of antidepressants, previous deliberate self-harm, pessimism, length of hospital stay, history of traumatic events, the level of social support, and the presence of depression, which was found to be a major determinant of SI in many studies [6, 10–14]. While clinical depression was found to be a common factor related to SI in previous research and this study, the dissimilarity of the results between both prior studies [1, 5] and current one may be caused by various sample sizes of inpatients identified with SI and different variables in the analysis, for example, the physical domain of QOL which was not explored in the other two studies but seemed to play a role in the presence of SI in our study.

### The association of QOL and SI

Our finding is consistent with that of a study that revealed that a lower level of QOL was correlated with SI in the elderly [6]. A study that enrolled 4506 adults in the community aged 60 or above indicated an inverse relationship between the levels of QOL with SI [7]. After adjustment for covariates, our study found that the physical domain of WHOQOL-BREF was correlated with SI among elderly inpatients. In addition to “satisfaction with sleep”, further analysis indicated “mobility” was the major determinants of QOL associated with SI. A large survey of 8500 adults over the age of 65, which found a moderate limitation in usual activities caused by arthritis and renal failure increased the risk of suicidal ideation and suicide attempt, supports our findings [45]. Additionally, a systematic analysis has shown the association between physical illness/functional disability and suicidal behavior [46].

### Clinical implication based on the analysis of items of BSRS-5 and QOL-PHY related to SI

In short, the analysis of all factors revealed that depression, inferiority, insomnia in BSRS-5, and mobility in QOL-PHY were associated with the presence of SI. On the basis of findings from our study and prior research, it is important to screen elderly inpatients with depression, insomnia, or feelings of inferiority to others, and provide support for exploring the potentially effective treatment of these conditions to lessen the risk of SI and subsequent suicide attempt. In addition, it is necessary to remind the medical staff to improve the mobility and activities for physically ill elderly inpatients, particularly when they have presented with aforementioned BSRS-5 risk factors for SI. It is suggested that a suicide-intervention support team be setup in the hospital, especially focusing on inpatients with mobility limitations. Early screening and adequate intervention, such as using mobility aids, providing transport assistance, maximizing involvement in recreational activities, and arranging rehabilitation programs or surgical procedures improving mobility, such as arthroplasty for this population, are expected to decrease the suicidal ideation and suicidal behavior in the future.

### Related factors of MTS-WTD in patients with SI

Among the elderly receiving primary care services, the prevalence of WTD was 6% [47]. Most strikingly, WTD was found to be associated with 5-year mortality independently of depressive status in the elderly in primary care patients who had a WTD without depression still had a higher mortality rate than those without WTD [9]. In other similar studies, the prevalence of inpatients with WTD seemed to be much lower than those with SI [1, 5]. Different from the analysis of prior studies, which evaluated the prevalence of all participants, our study, which revealed that 25.4% of patients with SI had a WTD, only assessed the presence of WTD for suicidal ideators. A study exploring the motives for suicide found that suicide attempts could be categorized into WTD and wish to change (WTC); the WTD group rather than the WTC group had a higher risk for suicide death [48]. Based on the observation of prior studies and our finding that only one-fourth of suicidal ideators had a MTS-WTD, it is reasonable to postulate that suicidal ideators who had a WTD might have a higher risk for subsequent suicidal behavior than those who had SI alone, although this hypothesis was not tested in the current study.

Our univariate analysis found that living alone, the level of negative feeling, and the level of being accepted/respected were associated with MTS-WTD in participants with SI. This observation is in accordance with that of a prior study, which revealed that depression and pessimism were related to WTD in medically ill elderly inpatients [5]. Likewise, other studies support our finding: the status of living was associated with suicide in the elderly [18]; perceived sense of belonging, substantial support, self-esteem, and chronic interpersonal problems were correlated with SI [49]; the factors related to WTD in the elderly were depression [50], hopelessness [51], social support, and subjective well-being [52]. The significant association between WTD and three variables in our study, i.e., living alone, negative feeling, and feelings of being accepted/respected suggest medical staff take proper measure in time; that is, when elderly inpatients are found to live alone before admission, or to present with negative feelings, such as hopelessness or a sense of being abandoned, they should be evaluated for the presence of WTD. If the above factors related to WTD are confirmed in an individual, for the purpose of preventing suicide, it should be recommended that they consult a social worker to provide sufficient social support, or a psychiatrist for proper evaluation and treatment, such as prescribing psychotropic agents, referral for psychological counseling, or administering cognitive behavior therapy. It is worth noting that WTD is an important issue in terminally ill patients in palliative care, although we did not address this topic here. There were many reasons when patients expressed a WTD [53]. It is of importance to broaden therapeutic options for suicide prevention and intervention by detecting the presence of WTD and appraising the motives for WTD statement in elderly inpatients.

### Models of BSRS-5 predicting SI

Our study showed that the PSM including depression, inferiority, and insomnia was a parsimonious model predictive of SI compared to the 5/6 for BSRS-5 model and the other model including all five BSRS-5 items. This finding has double meanings: (1) depression, inferiority, and insomnia are the most important items of the BSRS-5, explaining the variance of SI in the physically ill elderly inpatients; (2) the predictive value of a PSM made up of three items of BSRS-5 on SI is better than that of a model using a single variable of BSRS total scores greater than 5 alone; this implies that not all items of the BSRS-5 contribute equally or significantly to the presence of SI. Our result revealed that there was a significant association between three items of BSRS-5, i.e. depression, inferiority, and insomnia and the presence of SI. However, limited by the cross-sectional design of this study, it does not necessary mean that SI causes the presence of these three items; it could also be that depression, inferiority, and insomnia are the results of SI. Of note, this finding highlights a fact that these three items of BSRS-5 are important markers for patients with SI. It is necessary to screen elderly inpatients presenting with these items and to tailor specific interventions for maximizing the effectiveness of suicidal prevention under the limited resources. A further implication is to use the PSM, including three items of the BSRS-5, to single out those with SI in this population (please download the calculator presented in the form of Excel [54]) although the calculation of the BSRS-5 total score for a cut-off point is seemingly easier to use.

This result is partly in line with that of a study also using the BSRS-5 which revealed that depression, inferiority, insomnia, and hostility were significantly related to the presence of SI in 969 hospitalized patients with general medical conditions in southern Taiwan [24]. Different from that study, hostility was not associated with SI in the current study. This disparity in results between the former study and our study may be due in part to dissimilarities in following factors: (1) age group: not specifying detail information of age in the former vs. elderly adults in our study, (2) the prevalence of SI: 12.7% (123/969) vs. 3.1% (68/2199), and (3) the mean score of hostility: 0.74 vs. 0.65. The most remarkable evidence indicating the characteristics of SI in elderly inpatients which differed from other age groups, is that the former study proposed a parsimonious model using BSRS-5 total scores 12/13 as a cut-off point predictive of SI (PPV = 0.92 and NPV = 0.88). However, this cut-off point tested in our study was found to be a statistically insignificant model for predicting SI in elderly inpatients (PPV = 0.95, NPV =0.52, AUC = 0.55, *p* = 0.115). This finding indicates that physically ill elderly inpatients have unique features regarding SI, which may be distinctly different from those of other age groups. More studies exploring the age effect on SI among inpatients with medical and surgical conditions are needed in the future.

### Strength and limitation

To our knowledge, the strength of this study is that it is the first one recruiting a very large sample size of participants to explore SI, WTD, and QOL in physically ill elderly inpatients in East Asia. Second, a PSM was developed for more accurately predicting the presence of SI in this population. One possible limitation of this study is that the authors did not explore other factors related to the SI, such as the burden of physical illness, history of traumatic events, and substance misuse. Second, we only surveyed MTS-WTD in patients who had SI rather than all participants, and used univariate rather than multivariate analysis to explore the factors correlated to MTS-WTD owing to a small size of patients who had a MTS-WTD. Additionally, for establishing a predictive model, we did not have another large sample size of elderly inpatients as a validation group to retest the predictivity. Lastly, a very important limitation is that our study is cross-sectional; hence, causal inference between outcomes and risk factors should be made with caution.

In conclusion, in comparison with a traditional model using the BSRS-5 total score 5/6 as a cut-off point, we found that a propensity score model comprising three items of the BSRS-5, i.e., depression, insomnia, and inferiority are associated with the presence of SI among physically ill elderly inpatients. In addition to these three items, mobility in the WHOQOL-BREF is also correlated with SI. Approximately one-fourth of suicidal ideators had a MTS-WTD, which is associated with living alone, negative feelings, and feelings of not being accepted. WTD should be seen as a signal of a higher risk for subsequent suicidal behavior among those with SI. Careful appreciation of meanings and reasons for WTD for an individual is necessary to develop a mixture of effective therapeutic options for suicide prevention. To provide physical recovery and maintain mental health for physically ill elderly inpatients, setting up a multi-faceted approach targeting the aforementioned determinants of SI and WTD for reducing the risk of suicide attempts, and exploring other factors correlated with suicidal behaviors, are important topics and directions for clinical practice and further research.

## Abbreviations

BSRS-5: The 5-item brief symptom rating scale
BSSI: The Beck Scale for suicidal ideation
MMSE: The mini-mental state examination
MTS-WTD: Moderate to strong wish to die
PS: Propensity score
PSM: Propensity score model
QOL: Quality of life
QOL-ENV: The domain of environment of the Taiwanese version of World Health Organization Quality of Life-BREF
QOL-PHY: The domain of physical health of the Taiwanese version of World Health Organization Quality of Life-BREF
QOL-PSY: Psychological domain of the Taiwanese version of World Health Organization Quality of Life-BREF
QOL-SR: The domain of social relationships of the Taiwanese version of World Health Organization Quality of Life-BREF
SI: Suicidal ideation
WHOOQOL: The World Health Organization Quality of Life
WHOQOL-BREF TW: The Taiwanese version of World Health Organization Quality of Life-BREF
WTD: Wish to die

## References

[1] Ekramzadeh S, Javadpour A, Draper B, Mani A, Withall A, Sahraian A (2012). Prevalence and correlates of suicidal thought and self-destructive behavior among an elderly hospital population in Iran. Int Psychogeriatr.

[2] De Leo D, Padoani W, Lonnqvist J, Kerkhof AJ, Bille-Brahe U, Michel K, Salander-Renberg E, Schmidtke A, Wasserman D, Caon F (2002). Repetition of suicidal behaviour in elderly Europeans: a prospective longitudinal study. J Affect Disord.

[3] Stillion JM, McDowell EE (1991). Examining suicide from a life span perspective. Death Stud.

[4] Callahan CM, Hendrie HC, Nienaber NA, Tierney WM (1996). Suicidal ideation among older primary care patients. J Am Geriatr Soc.

[5] Shah A, Hoxey K, Mayadunne V (2000). Suicidal ideation in acutely medically ill elderly inpatients: prevalence, correlates and longitudinal stability. Int J Geriatr Psychiatry.

[6] Ponte C, Almeida V, Fernandes L (2014). Suicidal ideation, depression and quality of life in the elderly: study in a gerontopsychiatric consultation. Span J Psychol.

[7] Kim JH, Kwon JW (2012). The impact of health-related quality of life on suicidal ideation and suicide attempts among Korean older adults. J Gerontol Nurs.

[8] Palacio-Acosta C, Garcia-Valencia J, Diago-Garcia J, Zapata C, Ortiz-Tobon J, Lopez-Calle G, Lopez-Tobon M (2005). Characteristics of people committing suicide in Medellin, Colombia. Rev Salud Publica (Bogota).

[9] Raue PJ, Morales KH, Post EP, Bogner HR, Have TT, Bruce ML (2010). The wish to die and 5-year mortality in elderly primary care patients. Am J Geriatr Psychiatry.

[10] Liu IC, Chiu CH (2009). Case-control study of suicide attempts in the elderly. Int Psychogeriatr.

[11] Chen CS, Yang MS, Yang MJ, Chang SJ, Chueh KH, Su YC, Yu CY, Cheng TC (2008). Suicidal thoughts among elderly Taiwanese aboriginal women. Int J Geriatr Psychiatry.

[12] Yen YC, Yang MJ, Yang MS, Lung FW, Shih CH, Hahn CY, Lo HY (2005). Suicidal ideation and associated factors among community-dwelling elders in Taiwan. Psychiatry Clin Neurosci.

[13] Awata S, Seki T, Koizumi Y, Sato S, Hozawa A, Omori K, Kuriyama S, Arai H, Nagatomi R, Matsuoka H (2005). Factors associated with suicidal ideation in an elderly urban Japanese population: a community-based, cross-sectional study. Psychiatry Clin Neurosci.

[14] Yip PS, Chi I, Chiu H, Chi Wai K, Conwell Y, Caine E (2003). A prevalence study of suicide ideation among older adults in Hong Kong SAR. Int J Geriatr Psychiatry.

[15] Cheung YB, Law CK, Chan B, Liu KY, Yip PS (2006). Suicidal ideation and suicidal attempts in a population-based study of Chinese people: risk attributable to hopelessness, depression, and social factors. J Affect Disord.

[16] Draper BM (2014). Suicidal behaviour and suicide prevention in later life. Maturitas.

[17] Goodwin RD, Marusic A (2011). Perception of health, suicidal ideation, and suicide attempt among adults in the community. Crisis.

[18] De Leo D, Padoani W, Scocco P, Lie D, Bille-Brahe U, Arensman E, Hjelmeland H, Crepet P, Haring C, Hawton K (2001). Attempted and completed suicide in older subjects: results from the WHO/EURO multicentre study of suicidal behaviour. Int J Geriatr Psychiatry.

[19] Dombrovski AY, Butters MA, Reynolds CF, Houck PR, Clark L, Mazumdar S, Szanto K (2008). Cognitive performance in suicidal depressed elderly: preliminary report. Am J Geriatr Psychiatry.

[20] Marzuk PM, Hartwell N, Leon AC, Portera L (2005). Executive functioning in depressed patients with suicidal ideation. Acta Psychiatr Scand.

[21] Juurlink DN, Herrmann N, Szalai JP, Kopp A, Redelmeier DA (2004). Medical illness and the risk of suicide in the elderly. Arch Intern Med.

[22] Shah A, Hoxey K, Mayadunne V (2000). Some predictors of mortality in acutely medically ill elderly inpatients. Int J Geriatr Psychiatry.

[23] Lee MB, Liao SC, Lee YJ, Wu CH, Tseng MC, Gau SF, Rau CL (2003). Development and verification of validity and reliability of a short screening instrument to identify psychiatric morbidity. J Formos Med Assoc.

[24] Lung FW, Lee MB (2008). The five-item Brief-symptom rating scale as a suicide ideation screening instrument for psychiatric inpatients and community residents. BMC Psychiatry.

[25] Chen HC, Wu CH, Lee YJ, Liao SC, Lee MB (2005). Validity of the five-item Brief symptom rating scale among subjects admitted for general health screening. J Formos Med Assoc.

[26] Chen WJ, Chen CC, Ho CK, Lee MB, Chung YT, Wang YC, Lin GG, Lu RY, Sun FC, Chou FH (2009). The suitability of the BSRS-5 for assessing elderly who have attempted suicide and need to be referred for professional mental health consultation in a metropolitan city, Taiwan. Int J Geriatr Psychiatry.

[27] Wu CY, Lee JI, Lee MB, Liao SC, Chang CM, Chen HC, Lung FW (2016). Predictive validity of a five-item symptom checklist to screen psychiatric morbidity and suicide ideation in general population and psychiatric settings. J Formos Med Assoc.

[28] Shaffer D, Scott M, Wilcox H, Maslow C, Hicks R, Lucas CP, Garfinkel R, Greenwald S (2004). The Columbia suicide screen: validity and reliability of a screen for youth suicide and depression. J Am Acad Child Adolesc Psychiatry.

[29] Beck AT, Kovacs M, Weissman A (1979). Assessment of suicidal intention: the scale for suicide ideation. J Consult Clin Psychol.

[30] Yao G, Chung CW, Yu CF, Wang JD (2002). Development and verification of validity and reliability of the WHOQOL-BREF Taiwan version. J Formos Med Assoc.

[31] Yao G, Wang JD, Chung CW (2007). Cultural adaptation of the WHOQOL questionnaire for Taiwan. J Formos Med Assoc.

[32] Starcevic V, Bogojevic G, Marinkovic J (2000). The SCL-90-R as a screening instrument for severe personality disturbance among outpatients with mood and anxiety disorders. J Personal Disord.

[33] Guo NW, Liu HC, Wong PF, Liao KK, Yan SH, Lin KP, Chang CY, Hsu TC (1988). Chinese version and norms of the mini-mental state examination. J Rehabil Med.

[34] Folstein MF, Folstein SE, McHugh PR (1975). "Mini-mental state". A practical method for grading the cognitive state of patients for the clinician. J Psychiatr Res.

[35] Williamson EJ, Forbes A (2014). Introduction to propensity scores. Respirology.

[36] Hanley JA, McNeil BJ (1983). A method of comparing the areas under receiver operating characteristic curves derived from the same cases. Radiology.

[37] Richardson JD, Thompson A, King L, Corbett B, Shnaider P, St Cyr K, Nelson C, Sareen J, Elhai J, Zamorski M (2017). Insomnia, psychiatric disorders and suicidal ideation in a National Representative Sample of active Canadian forces members. BMC Psychiatry.

[38] Michaels MS, Balthrop T, Nadorff MR, Joiner TE (2017). Total sleep time as a predictor of suicidal behaviour. J Sleep Res.

[39] Zuromski KL, Cero I, Witte TK (2017). Insomnia symptoms drive changes in suicide ideation: a latent difference score model of community adults over a Brief interval. J Abnorm Psychol.

[40] Escobar-Cordoba F, Quijano-Serrano M, Calvo-Gonzalez JM (2017). Evaluation of insomnia as a risk factor for suicide. Rev Fac Cien Med Univ Nac Cordoba.

[41] Moritz S, Werner R, von Collani G (2006). The inferiority complex in paranoia readdressed: a study with the implicit association test. Cogn Neuropsychiatry.

[42] Perrot C, Vera L, Gorwood P (2018). Poor self-esteem is correlated with suicide intent, independently from the severity of depression. Encephale.

[43] Bagalkot TR, Park JI, Kim HT, Kim HM, Kim MS, Yoon MS, Ko SH, Cho HC, Chung YC (2014). Lifetime prevalence of and risk factors for suicidal ideation and suicide attempts in a Korean community sample. Psychiatry.

[44] Dieserud G, Roysamb E, Ekeberg O, Kraft P (2001). Toward an integrative model of suicide attempt: a cognitive psychological approach. Suicide Life Threat Behav.

[45] Kim SH (2016). Suicidal ideation and suicide attempts in older adults: influences of chronic illness, functional limitations, and pain. Geriatr Nnurs.

[46] Fassberg MM, Cheung G, Canetto SS, Erlangsen A, Lapierre S, Lindner R, Draper B, Gallo JJ, Wong C, Wu J (2016). A systematic review of physical illness, functional disability, and suicidal behaviour among older adults. Aging Ment Health.

[47] Kim YA, Bogner HR, Brown GK, Gallo JJ (2006). Chronic medical conditions and wishes to die among older primary care patients. Int J Psychiatry Med.

[48] Unni KE, Rotti SB, Chandrasekaran R (1995). An exploratory study of the motivation in suicide attempters. Indian J Psychiatry.

[49] Harrison KE, Dombrovski AY, Morse JQ, Houck P, Schlernitzauer M, Reynolds CF, Szanto K (2010). Alone? Perceived social support and chronic interpersonal difficulties in suicidal elders. Int Psychogeriatr.

[50] Barnow S, Linden M (2002). Psychosocial risk factors of the wish to be dead in the elderly. Fortschr Neurol Psychiatr.

[51] Cukrowicz KC, Jahn DR, Graham RD, Poindexter EK, Williams RB (2013). Suicide risk in older adults: evaluating models of risk and predicting excess zeros in a primary care sample. J Abnorm Psychol.

[52] Bonnewyn A, Shah A, Bruffaerts R, Demyttenaere K (2017). Factors determining the balance between the wish to die and the wish to live in older adults. Int J Geriatr Psychiatry.

[53] Ohnsorge K, Gudat H, Rehmann-Sutter C (2014). What a wish to die can mean: reasons, meanings and functions of wishes to die, reported from 30 qualitative case studies of terminally ill cancer patients in palliative care. BMC Palliat Care.

[54] The Database for predicting suicidal ideation. 2017. https://www.dropbox.com/s/mywp9hxohxqp1k4/ROC%20computer%20-bsrs1-5.xls?dl=0.

